# A modified Delphi study of screening for fetal alcohol spectrum disorders in Australia

**DOI:** 10.1186/1471-2431-13-13

**Published:** 2013-01-25

**Authors:** Rochelle E Watkins, Elizabeth J Elliott, Jane Halliday, Colleen M O’Leary, Heather D’Antoine, Elizabeth Russell, Lorian Hayes, Elizabeth Peadon, Amanda Wilkins, Heather M Jones, Anne McKenzie, Sue Miers, Lucinda Burns, Raewyn C Mutch, Janet M Payne, James P Fitzpatrick, Maureen Carter, Jane Latimer, Carol Bower

**Affiliations:** 1 Telethon Institute for Child Health Research, Centre for Child Health Research, The University of Western Australia, Perth, Australia; 2 Discipline of Paediatrics and Child Health, Sydney Medical School, University of Sydney, Sydney, Australia; 3 The Children’s Hospital at Westmead, Sydney, Australia; 4 The George Institute for Global Health, Sydney, Australia; 5 Public Health Genetics, Genetic Disorders, Murdoch Childrens Research Institute, Melbourne, Australia; 6 Department of Paediatrics, University of Melbourne, Melbourne, Australia; 7 Centre for Population Health Research, Curtin University, Perth, Australia; 8 Menzies School of Health Research, Charles Darwin University, Darwin, Australia; 9 Russell Family Fetal Alcohol Disorders Association, Cairns, Australia; 10 Centre for Chronic Disease, School of Medicine, University of Queensland, Brisbane, Australia; 11 Child and Adolescent Health Service, Department of Health Western Australia, Perth, Australia; 12 National Organisation for Fetal Alcohol Syndrome and Related Disorders, Adelaide, Australia; 13 National Drug and Alcohol Research Centre, University of New South Wales, Sydney, Australia; 14 Nindilingarri Cultural Health Services, Fitzroy Crossing, Australia

## Abstract

**Background:**

There is little reliable information on the prevalence of fetal alcohol spectrum disorders (FASD) in Australia and no coordinated national approach to facilitate case detection. The aim of this study was to identify health professionals’ perceptions about screening for FASD in Australia.

**Method:**

A modified Delphi process was used to assess perceptions of the need for, and the process of, screening for FASD in Australia. We recruited a panel of 130 Australian health professionals with experience or expertise in FASD screening or diagnosis. A systematic review of the literature was used to develop Likert statements on screening coverage, components and assessment methods which were administered using an online survey over two survey rounds.

**Results:**

Of the panel members surveyed, 95 (73%) responded to the questions on screening in the first survey round and, of these, 81 (85%) responded to the second round. Following two rounds there was consensus agreement on the need for targeted screening at birth (76%) and in childhood (84%). Participants did not reach consensus agreement on the need for universal screening at birth (55%) or in childhood (40%). Support for targeted screening was linked to perceived constraints on service provision and the need to examine the performance, costs and benefits of screening.

For targeted screening of high risk groups, we found highest agreement for siblings of known cases of FASD (96%) and children of mothers attending alcohol treatment services (93%). Participants agreed that screening for FASD primarily requires assessment of prenatal alcohol exposure at birth (86%) and in childhood (88%), and that a checklist is needed to identify the components of screening and criteria for referral at birth (84%) and in childhood (90%).

**Conclusions:**

There is an agreed need for targeted but not universal screening for FASD in Australia, and sufficient consensus among health professionals to warrant development and evaluation of standardised methods for targeted screening and referral in the Australian context. Participants emphasised the need for locally-appropriate, evidence-based approaches to facilitate case detection, and the importance of ensuring that screening and referral programs are supported by adequate diagnostic and management capacity.

## Background

Prenatal alcohol exposure causes a range of disorders of fetal development [1-3]. The term fetal alcohol spectrum disorders (FASD) is used to identify one group of conditions that can result from prenatal exposure to alcohol [4]. Fetal alcohol spectrum disorders feature characteristic combinations of facial anomalies, growth impairment, intellectual disability, behavioural disorders and birth defects [5-8], although the spectrum of disorders most commonly includes impairments of neurological function that are not accompanied by characteristic facial anomalies or growth deficit [9,10]. There is some variation in the range of disorders recognised within the spectrum and the use of specific diagnostic terminology between different diagnostic guidelines. However, all guidelines include the well-established diagnostic category of fetal alcohol syndrome (FAS) [6,8,11-13], and most include diagnostic categories for partial FAS (PFAS), and alcohol-related neurodevelopmental disorder (ARND) or its equivalent [6,8,11,13].

The diagnosis of FASD is often delayed or missed [4,14-16], despite evidence linking diagnosis and intervention to improved educational, social and health outcomes [17,18]. Failure to identify children with FASD has been attributed to the limited ability of health service providers to access affected children and their mothers, a lack of accurate routine screening for FASD or of routine screening for maternal alcohol use during pregnancy, limited clinical capacity to recognise and diagnose these disorders, and the difficulty of identifying all but the most severe cases at birth [19,20]. The identification of FASD in Australia is likely to be limited by poor awareness of diagnostic features among paediatricians [21] and other health professionals [22], and the absence of national guidelines for diagnosis. Concern about stigma as a result of diagnosis [22] may also influence the willingness to diagnose or to be diagnosed.

There is only limited information available on the prevalence of FASD in Australia [15], with no routine screening or surveillance established for the disorders. The need to address significant gaps in the capacity to identify and diagnose FASD in Australia is reflected in recent health policy [23] and the launch of an Australian federal government inquiry into the incidence and prevention of FASD in 2011 [24]. Although not yet implemented, the Western Australian FASD Model of Care [23] recommends multi-stage population screening to identify at risk newborns and children based on history of maternal alcohol consumption, abnormal growth parameters and developmental delay. Targeted screening is also recommended in a number of sub-groups considered at high risk, including children of mothers who receive alcohol treatment services and children in state care.

Screening for FASD provides a means to facilitate referral, diagnosis and support [6]; however, several factors complicate the identification of, and screening for, FASD [12]. Screening for FAS has primarily utilised identification of the three characteristic FAS facial anomalies (short palpebral fissures, smooth philtrum and thin upper lip) that are collectively specific to FAS [25]; although routinely collected data from neonatal medical records including growth, head circumference, maternal alcohol use and other criteria have also been used [26]. Screening for FAS using facial photographs to detect the characteristic facial anomalies has been found to be effective in high-risk populations in North America, with a positive predictive value of 86% and a negative predictive value of 100% [27].

Screening for FASD is more difficult because facial dysmorphology is frequently absent, impairments of neurological function are predominant [6], and the neurobehavioural features are not unique to FASD [28]. Inconsistency in the methods used for screening and referral internationally [29] is linked to a lack of screening instruments for FASD that are specific and sensitive to the fetal effects of prenatal alcohol exposure [6]. Biochemical screening has the potential to facilitate early diagnosis; however, apart from the detection of ethyl esters in newborn meconium, screening methods are not well established [30,31]. Heterogeneity among the observed features of FASD due to variation in genetic factors, patterns of alcohol exposure and other maternal risk factors adds to the complexity of screening for FASD [29]. Nevertheless, the use of standard screening processes may facilitate the appropriate identification of individuals who require specialist diagnostic assessment.

Screening programs for both FAS and FASD frequently use assessment methods that are directly linked to diagnostic criteria. Published screening assessments have included prenatal alcohol exposure, weight, height, head circumference, developmental delay, learning difficulties, behavioural problems and the presence of characteristic FAS facial anomalies [9,26,32]. However, screening programs do not always assess the full range of diagnostic features. For example, facial dysmorphology assessment has been excluded from FASD screening criteria [9,32], consistent with the recommended need for experienced assessors, the infrequent occurrence of facial anomalies among individuals with FASD, and the lack of norms for ethnically diverse populations [29].

There is little locally-relevant evidence on which to base policy decisions about FASD screening in Australia. There is no coordinated national approach to facilitate the identification of FASD in Australia, and it is unclear to what extent current policy and capacity for service delivery are consistent with clinician perspectives of priorities and needs. We aimed to evaluate perceptions about screening for FASD among Australian health professionals with experience or expertise in FASD screening or diagnosis to guide research and policy on FASD screening in Australia.

## Methods

A modified Delphi process [33] was used to assess Australian health professionals’ perceptions of the need for and process of FASD screening in Australia. The modified Delphi design generally diverges from the classical Delphi method in the use of alternative means to derive the content of the initial quantitative questionnaire round [33] while still allowing the collection of rich data based on multiple questionnaire iterations [34]. Modified Delphi studies are particularly appropriate where relevant knowledge exists.

### Questionnaire development

An existing systematic review on FASD screening and diagnosis [35] was used to identify literature relevant to the design of screening for FASD in Australia. The search strategy for this review was updated to include literature published up to and including September 2010, and expanded to include additional search terms (fetal alcohol effects, fetal alcohol disorders, partial fetal alcohol syndrome, alcohol related birth defects, alcohol related neurodevelopmental disorder, screen$, diagnos$). Additional relevant databases were also searched (CINAHL, PsychINFO and Web of Science).

Descriptions of screening programs identified in the published literature were used to design Likert statements to evaluate perceptions about screening coverage, screening components, and assessment methods. Participants were asked to rate their agreement with each statement on a 5-point Likert scale which ranged from ‘strongly agree’ to ‘strongly disagree’, and a response option of ‘no comment’ was provided to enable participants to indicate that a statement was outside their area of expertise. Each question area also included open ended questions and participants were encouraged to provide comments on their responses and identify where the statements did not reflect their beliefs. The questions on screening were administered as part of a larger survey on the screening and diagnosis of FASD in Australia. Perceptions about the diagnosis of FASD have been reported elsewhere [36]. The questionnaire was pilot tested with 16 health professionals and health researchers to examine coherence, feasibility and face validity prior to administration.

### Panel recruitment

We aimed to recruit a large panel of Australian health professionals with experience or expertise in FASD screening or diagnosis. Panel members were identified using three purposive sampling strategies [37]: i) medical practitioners who reported a diagnosis of FAS to the Australian Paediatric Surveillance Unit (APSU) in a previous study [15], ii) health professionals who were identified by study investigators as having experience or expertise in FASD screening or diagnosis, and iii) individuals who responded to calls to health professional organisations for individuals who had relevant experience or expertise.

Recruitment of panel members with relevant experience or expertise underpins the validity of this study, and processes used to enrol panel members varied by sampling strategy based on the need to confirm relevant expertise prior to enrolment. Information about this study was distributed to 57 eligible medical practitioners who had reported a diagnosis of FAS to the APSU, and all were included in the study apart from 17 who either actively declined to participate or who could not be contacted (based on an invalid email address or an automated email reply). In contrast, all other invited health professionals were only included in the panel if they actively confirmed their suitability and willingness to participate. Among these 149 invited health professionals, 90 accepted the invitation to participate. The modified Delphi process commenced with 130 Australian panel members: including 40 who had reported a diagnosis of FAS to the APSU, 59 who were identified as having experience or expertise in FASD screening or diagnosis, and 31 who were recruited from professional organisations.

### Questionnaire administration

The online password-protected questionnaire was administered from a secure web server, and responses were automatically saved to a secure MySQL database. All panel members were invited by email to complete the first round questionnaire within 14 days, and two email reminders were sent prior to the round deadline. Due to requests for a longer period for response, the round 1 deadline was extended by 8 days, and 21 days was provided for response to the round 2 questionnaire. Where contact details were available, panel members who had not visited the study website were targeted for additional follow-up. One contact attempt was made by telephone at least 5 days before the closure date of rounds 1 and 2. Participants who did not complete round 1 were excluded from round 2.

### Round 2 questionnaire revision

Both quantitative and qualitative responses were considered in the questionnaire revision process. Due to the length of the round 1 questionnaire, revisions aimed to minimise the length of the round 2 questionnaire and exclude question areas that had achieved consensus. If at least 70% of participants agreed or strongly agreed with a statement, it was considered endorsed. This level of consensus was decided *a priori*. If fewer than 60% of participants agreed or strongly agreed with a statement, the statement was rejected or modified based on qualitative findings. However, where multiple statements were related to the same issue, consensus was sought for the question area rather than for each statement in isolation. As such, several statements that either achieved consensus agreement or less than 60% agreement were included in round 2 where clear consensus was not achieved for that question area. New statements were also included in round 2 if qualitative findings indicated that the round 1 questionnaire failed to represent a relevant alternative perspective. The round 2 questionnaire included feedback of representative comments as well as group and individual per cent agreement results from round 1 to enable participants to reflect on their previous responses.

### Analysis

Descriptive statistics were generated for each statement, including response frequencies and dispersion (inter-quartile deviation). Associations between ratings for key statements on screening coverage and individual characteristics, were explored using the Chi square test or Fisher’s exact test [38]. The Wilcoxon signed rank test was used to compare agreement between statements on screening coverage, and between alternative screening methods for growth. All analyses were evaluated using two-tailed test statistics.

Qualitative data were coded and analysed independently by two investigators using an inductive content analysis approach [39,40]. Data from each open ended question were reviewed alongside the quantitative data, compared and coded based on the underlying meaning of the responses. Both analysts’ coding schemes were reviewed for consistency to ensure credibility and trustworthiness of the analysis process [39]. This study was approved by the University of Western Australia Human Research Ethics Committee and the Western Australian Aboriginal Health Information and Ethics Committee.

## Results

Of the 130 panel members, 95 (73%) responded in round 1, and 81 of these 95 (85%) responded in round 2. Response in round 1 was: 68% (27/40) among panel members who reported a diagnosis of FAS to the APSU; 78% (46/59) among panel members recruited by the study investigators; and 71% (22/31) among panel members recruited through professional organisations. Round 1 participants were most commonly paediatricians (46%), and approximately three quarters reported experience in FASD screening or diagnosis (Table 1).

**Table 1:** Summary of participant characteristics in round 1

**Characteristic**	**n (%) (n=95)**
Sex	
Male	24 (25)
Female	71 (75)
State^†^	
Australian Capital Territory	1 (1)
New South Wales	23 (26)
Northern Territory	5 (6)
Queensland	23 (26)
South Australia	6 (7)
Tasmania	3 (3)
Victoria	4 (5)
Western Australia	24 (27)
Occupation	
Paediatricians	44 (46)
Non-paediatrician medical practitioners	22 (23)
Other health professionals^Δ^	29 (31)
Experience in FASD diagnosis^‡^	
Yes	38 (41)
No	55 (59)
Experience in FASD screening, diagnosis or contributing^*^ to diagnosis^‡^	
Yes	70 (75)
No	23 (25)
Completed specific training on FASD diagnosis^‡^	
Yes	20 (22)
No	73 (79)
Work location^‡^	
Includes rural or remote practice	41 (44)
Does not include rural or remote practice	52 (56)

^†^Valid n = 89.

^‡^Valid n = 93.

^Δ^Other health professionals includes nurses, allied health professionals, health workers and health researchers.

^*^Contribution to diagnosis includes the conduct of relevant assessments that inform diagnosis, but not determination of the final diagnosis.

### Screening coverage

Due to a lack of consensus on the use of targeted screening at birth following round 1, all four statements evaluating screening coverage were re-administered in round 2 (Table 2, statements 1-4). After round 2 there was no strong evidence of a difference in agreement that screening for FASD at birth should be targeted (76%) or universal (55%; Wilcoxon Z=-1.7, p=0.09). Participants most commonly agreed that screening for FASD in childhood should be targeted (84%), and were more likely to agree that it should be targeted than universal (40%; Wilcoxon Z=-4.1, p<0.001). They were also more likely to agree with the use of targeted screening in childhood compared with targeted screening at birth (Wilcoxon Z=-2.2, p=0.03). We found no evidence of differences in agreement about screening coverage at birth or in childhood according to experience in screening or diagnosis (all p>0.7).

**Table 2:** Agreement with statements on screening coverage and indications in rounds 1 and 2

**Statement**	**R1 N**	**R1 % Agree**^**† **^**(IQD)**	**R2 % Agree**^**† **^**(IQD)**
***Screening coverage***			
1. Screening for FASD at birth should be universal	87	58 (3)	55 (3)
2. Screening for FASD at birth should be targeted	88	68 (2)	**76 (1)**
3. Screening for FASD in childhood should be universal	86	49 (3)	40 (2)
4. Screening for FASD in childhood should be targeted	86	**78 (1)**	**84 (1)**
***Indications for targeted screening - presentations***			
5. an alcohol-related event, illness or dependency in the birth mother	91	**96 (1)**	-
6. a parent/foster parent who is concerned that their child might have a FASD	91	**99 (1)**	-
7. prenatal alcohol exposure	90	**92 (1)**	-
8. developmental delay	88	**91 (1)**	-
9. growth retardation or failure to thrive	87	**91 (1)**	-
10. structural central nervous system abnormalities	82	**87 (1)**	-
11. neurological signs	84	**82 (1)**	-
12. functional central nervous system abnormalities	84	**88 (1)**	-
13. characteristic FAS facial anomalies	89	**97 (1)**	-
14. birth defects	85	**93 (1)**	-
15. reported or observed problems with behaviour	88	**86 (1)**	-
***Indications for targeted screening – high risk groups***			
16. children of mothers attending alcohol treatment services	91	**93 (1)**	-
17. siblings of identified cases of FASD	90	**96 (1)**	-
18. children who are diagnosed with ADHD	82	**74 (2)**	-
19. children entering a child development service	89	**87 (1)**	-
20. children entering child protection	86	**85 (1)**	-
21. children entering foster care or adoptive placements (incl. kinship care)	86	**87 (1)**	-
22. children entering a juvenile justice setting	84	**82 (1)**	-

R1-Round 1; R2-Round 2; IQD-inter-quartile deviation.

^**†**^Includes responses ‘agree’ and ‘strongly agree.’

Results for statements that reached 70% agreement (consensus) are presented in bold.

There was clear consensus agreement on all indications for targeted screening evaluated in round 1 (Table 2, statements 5-22). Over 95% of participants agreed with screening for FASD where there is parental concern that their child may have a FASD; evidence of characteristic FAS facial anomalies; an alcohol-related illness or dependency in the birth mother; and a sibling with FASD.

In round 1, 61 participants (64%) provided comments about their preferences for screening coverage. Comments most frequently indicated support for universal screening as an ideal and ethical approach that decreases the risk of missed cases, helps overcome the limitations of a potentially unreliable maternal alcohol history, and enables early diagnosis and intervention. However, targeted screening of high risk groups was commonly supported as a more feasible and effective approach. Participants commonly identified practical constraints associated with screening and diagnosis, including the need for increased service capacity and provider training, the need for adequate early intervention services, and the evolving and variable nature of FASD presentations. Respondents also expressed concerns about the absence of well-established or accurate screening tests and the sensitivity, specificity and cost-effectiveness of screening. They also indicated support for the universal collection of a maternal history of alcohol use during the prenatal period and at birth to enable preventive interventions and targeted follow-up. Participants less commonly described adverse psychosocial consequences of screening or of assigning an aetiological diagnostic label such as maternal blame or stigma; and a lack of support for screening programs which focus on a single condition.

### Components of screening

Consensus was achieved for all components of screening assessed apart from the testing of fatty acid esters (Table 3, statements 1-8 and 12-21). Some participants commented in round 1 that the agreed components of screening for FASD were already routinely assessed at birth or during the assessment of neurodevelopmental problems presenting in childhood; that the primary need is for health professionals to assess prenatal alcohol exposure and consider it as a potential cause of identified abnormalities; and that a screening checklist and criteria for referral are required. Additional statements were included in the round 2 questionnaire to evaluate participants’ perceptions of these issues (Table 3, statements 9-11 and 22-24). Following round 2 there was no consensus agreement that information required for screening is currently routinely collected. However, there was consensus agreement that screening primarily requires health professionals to assess prenatal alcohol exposure and consider it as a potential cause of other relevant abnormalities identified at birth (86%) and in childhood (88%); and that a screening checklist is required to identify criteria for referral at birth (84%) and in childhood (90%).

**Table 3:** Agreement with statements on the components of screening at birth and in childhood in rounds 1 and 2

**Statement**	**R1 N**	**R1 % Agree**^**† **^**(IQD)**	**R2 % Agree**^**† **^**(IQD)**
***Screening at birth***			
1. prenatal alcohol exposure	92	**98 (1)**	-
2. birth weight, length and head circumference	90	**100 (1)**	-
3. fatty acid esters (FAEE) in meconium collected within 72 hours of birth	37	46 (3)	-
4. characteristic FAS facial anomalies	89	**98 (1)**	-
5. birth defects	89	**98 (1)**	-
6. evidence of withdrawal from alcohol or other drugs	90	**99 (1)**	-
7. family history of FASD or developmental delay	79	-	**95 (1)**
8. evidence of CNS dysfunction including irritability, feeding difficulties or other neurological signs	77		**91 (1)**
9. most of the information required for FASD screening at birth is routinely collected at birth	72	-	56 (2)
10. screening for FASD at birth primarily requires health professionals to assess prenatal alcohol exposure and consider it as a potential cause of other relevant abnormalities identified	79	-	**86 (1)**
11. a checklist is needed to support the implementation of screening for FASD at birth that identifies the components to be assessed and criteria for conducting a full diagnostic evaluation	79	-	**84 (1)**
***Screening in childhood***			
12. prenatal alcohol exposure	90	**97 (1)**	-
13. growth (height and weight)	89	**98 (1)**	-
14. head circumference	86	**99 (1)**	-
15. developmental delay	89	**99 (1)**	-
16. neurological signs	87	**93 (1)**	-
17. functional CNS abnormalities (e.g. cognition, behaviour disorders)	88	**99 (1)**	-
18.hearing and vision	85	**93 (1)**	-
19. characteristic FAS facial anomalies	89	**98 (1)**	-
20. birth defects	89	**97 (1)**	-
21. family history of FASD, developmental delay, abuse or neglect	78	-	**97 (1)**
22. most of the information required for FASD screening in childhood is routinely assessed as part of a general clinical assessment of children with neurodevelopmental or other related presentations	71	-	59 (2)
23. screening for FASD in childhood primarily requires health professionals to assess prenatal alcohol exposure and consider it as a potential cause of other relevant abnormalities identified (e.g. abnormalities of development, learning, behaviour)	77	-	**88 (1)**
24. a checklist is needed to support the implementation of screening for FASD in childhood that identifies the components to be assessed and criteria for conducting a full diagnostic evaluation	78	-	**90 (1)**

R1-Round 1; R2-Round 2; IQD-inter-quartile deviation; CNS-central nervous system.

^**†**^Includes responses ‘agree’ and ‘strongly agree.’

Results for statements that reached 70% agreement (consensus) are presented in bold.

Participant comments linked improved awareness of and ability to screen for FASD with improved case identification. Screening for prenatal alcohol exposure, although potentially unreliable, was considered by some participants to be the most important element of screening for FASD due to the difficulty of screening for the spectrum of disorders at birth, and the fact that central nervous system (CNS) dysfunction is often not apparent in very young children. Other participants referred to difficulties in screening associated with nondisclosure of alcohol use during pregnancy, or where no maternal history is available. Several participants also identified the limited usefulness of screening for facial anomalies when screening for FASD.

### Assessment methods

Almost all statements on screening assessment methods achieved consensus in round 1 (Table 4). There was a high level of agreement that the assessment of prenatal alcohol exposure should identify and record the number of standard drinks consumed on a typical drinking occasion, the frequency of drinking, the frequency of excessive drinking and the timing of alcohol intake during pregnancy (Table 4, statements 1-5). Most participants (71%) believed that prenatal alcohol exposure should be assessed using a formal tool (Table 4, statements 6-7).

**Table 4:** Agreement with statements on screening assessment methods for prenatal alcohol exposure, growth deficit and characteristic fetal alcohol syndrome facial anomalies in rounds 1 and 2

**Statement**	**R1 N**	**R1 % Agree**^**† **^**(IQD)**	**R2 % Agree**^**† **^**(IQD)**
***Prenatal alcohol exposure***			
Assessment of prenatal alcohol exposure should identify and record the:			
1. … number of standard drinks consumed during a typical drinking occasion	85	**98 (1)**	-
2. … frequency of drinking occasions	86	**98 (1)**	-
3. … frequency of excessive (binge) drinking (5+ standard drinks per occasion)	86	**95 (1)**	-
4. … timing of alcohol intake during pregnancy	86	**97 (1)**	-
5. Alcohol exposure should be assessed alongside other lifestyle factors (e.g. diet)	85	**92 (1)**	-
6. Prenatal alcohol exposure can be effectively assessed using an informal approach (e.g. inquiring during a consultation)	82	52 (2)	41 (2)
7. Prenatal alcohol exposure should be assessed using a formal tool	69	**71 (2)**	-
8. The use of formal tools for the assessment of prenatal alcohol exposure should be combined with a clinical interview to obtain more detailed information about alcohol consumption patterns, potential indicators of addiction and other relevant contextual information	80	-	**88 (1)**
9. Information on alcohol use from family members, other health professionals or community members (if appropriate) should be sought if indicated	78	-	**77 (0)**
10. The AUDIT-C would be a useful tool for the formal assessment of prenatal alcohol exposure	74	-	**89 (0)**
***Growth deficit***			
11. Growth should be assessed by comparing height and weight with population standards	70	**93 (1)**^1^	-
12. Growth should be assessed by comparing weight to height ratio with population standards	66	68 (2) ^1^	-
13. Growth should be assessed by comparing weights over time (to identify decelerating weight over time)	68	**90 (1) **^1^	-
14. Assessment of growth deficit should consider other factors that may affect growth (e.g. gestational age parental size, gestational diabetes, nutritional status, illness)	78	**100 (1)**	-
***Characteristic fetal alcohol syndrome (FAS) facial anomalies***			
15. The presence of the following characteristic FAS facial anomalies should be assessed: smooth philtrum, thin upper lip, and small palpebral fissures	81	**100 (1)**	-
16. Assessment of characteristic FAS facial anomalies should use appropriate anthropometric population standards for race and age where available	77	**95 (1)**	-
***At the screening stage, characteristic FAS facial anomalies can be effectively assessed using:***			
17. … clinical observation(R1) /Facial anomalies can be assessed using clinical observation for evidence of the characteristic FAS facial anomalies, with formal physical measurement of these features not essential at the screening stage (R2)	69	**73 (1)**	**77 (0)**
18. … physical measurement of palpebral fissures	50	**76 (0)**	-
19. … the University of Washington Lip-Philtrum Guide	49	**86 (1)**	-
20. … the facial photographic screening tool	45	**76 (1.5)**	-
21. Palpebral fissure length must be assessed using formal physical measurement and comparison with population references at the screening stage	62	**-**	39 (2)
22. Thin upper lip and smooth philtrum must be assessed using formal tools such as the University of Washington Lip-Philtrum Guide at the screening stage	61	**-**	46 (2)

R1-Round 1; R2-Round 2; IQD-inter-quartile deviation.

^**†**^Includes responses ‘agree’ and ‘strongly agree’.

Results for statements that reached 70% agreement (consensus) are presented in bold.

^1^Friedman test indicated a significant difference in agreement with the 3 statements that described different methods to assess growth (statements 11-13: Friedman chi-square=19.3, p<0.001). Post-hoc testing found a significant difference between ratings for statements 11 and 12 (Wilcoxon Z=-3.5, p<0.001) and 12 and 13 (Wilcoxon Z=-3.1, p=0.002).

The most commonly recommended formal assessment tool was the AUDIT-C (21%) [41], followed by Lifescripts (17%) [42] which incorporates the AUDIT-C, T-ACE (14%) and TWEAK (13%) [43]. Most participants reported that they were not familiar with the Lifescripts (63%), TWEAK (62%), T-ACE (62%) and AUDIT-C (59%) tools, and no alternative formal assessment tools were proposed. Following round 2, the role of informal inquiry in prenatal alcohol exposure assessment was clarified, with consensus agreement for the combination of informal and formal methods of inquiry. After receiving further information about the AUDIT-C in round 2, 89% of participants considered that it provided a useful screening tool for prenatal alcohol exposure (Table 4, statements 8-10).

Participants most frequently supported the comparison of height and weight with population standards to assess growth deficit (Table 4, statements 11-14), and there was a high level of consensus on the specific facial anomalies that should be assessed during screening (Table 4, statement 15). Participant comments in round 1 indicated a lack of distinction between screening and diagnostic assessments, the need for training to use facial anomaly assessment tools reliably, the lack of suitable Australian reference data for comparison, and the need for screening methods to be efficient. Following round 2 there was consensus agreement that formal assessment of facial anomalies could be used, but was not required, to assess facial anomalies at the screening stage (Table 4, statements 17-22).

There was agreement on the assessment of a broad range of CNS abnormalities in screening, and limited support for the use of brain imaging (Table 5, statements 1-14). Participant comments in round 1 highlighted the need to distinguish between screening and diagnostic assessments, and the need for a pragmatic approach to assessment considering cost and resources, including the possibility of qualitative assessment of core areas at the screening stage. Round 2 results indicated agreement that clinical identification or third party report were acceptable indicators of CNS abnormality at the screening stage (Table 5, statements 18-21).

**Table 5:** Agreement with statements on screening assessment methods for central nervous system abnormalities in rounds 1 and 2

**Statement**	**R1 N**	**R1 % Agree**^**† **^**(IQD)**	**R2 % Agree**^**† **^**(IQD)**
***Central nervous system (CNS) abnormalities***			
Assessment of CNS abnormalities in FASD screening may include:			
1. … developmental milestones	80	**96 (1)**	-
2. … motor and sensory function	77	**88 (1)**	-
3. … cognition (IQ)	79	**92 (1)**	-
4. … memory	78	**89 (1)**	-
5. … academic achievement	79	**91 (1)**	-
6. … executive functioning and abstract reasoning	79	**89 (1)**	-
7. … adaptive behaviour	75	**92 (1)**	-
8. … attention and hyperactivity	80	**95 (1)**	-
9. … communication (receptive and expressive language)	78	**94 (1)**	-
10. … social skills and social communication	80	**93 (1)**	-
11. … hard and soft neurologic signs (including sensory-motor signs)	74	**87 (1)**	-
12. … seizures that are not due to a postnatal insult or other postnatal process	74	**78 (1)**	-
13. … head circumference	78	**96 (1)**	-
14. … brain imaging	67	57 (1)	-
The choice of tests for neuro-behavioural assessments should be guided by:			
15. … the availability of valid and reliable instruments	76	**90 (1)**	-
16. … clinician preference and experience	74	60 (2) ^‡^	-
17. … test appropriateness for patient age and cultural background	78	**97 (1)**	-
18. At the screening stage it is not necessary to formally assess and measure suspected CNS anomalies	68	-	**72 (1)**
At the screening stage the following are acceptable indicators of possible CNS abnormalities (neurological, functional or structural):			
19. … clinical identification	66	-	**97 (0)**
20. … parent or other credible third party report	66	-	**91 (0)**
21. … results of previous relevant formal assessments (e.g. psychological report)	65	-	**100 (1)**
***Birth defects***			
22. FASD screening should assess and record the presence of birth defects as part of the clinical examination	81	**99 (1)**	-

R1-Round 1; R2-Round 2; IQD-inter-quartile deviation.

^**†**^Includes responses ‘agree’ and ‘strongly agree’.

^‡^Statement excluded from round 2 as proportion agreement was 59.5%.

Results for statements that reached 70% agreement (consensus) are presented in bold.

### Response completeness

Over half of the 95 participants (57%) completed 10 or fewer of the round 1 Likert statements on screening. Non-complete responses most commonly included a response of ‘no comment’, indicating that the statements were outside participant’s areas of expertise. Completion of 10 or fewer statements was more frequent among participants who reported no experience in diagnosis or screening (87%) and health professionals other than paediatricians (73%) than among participants who reported experience in diagnosis or screening (49%, χ^2^=11.7, p=0.001) and paediatricians (39%, χ^2^=11.3, p=0.001) respectively.

Assessment of the association between response completeness and perceptions found clear evidence of an association with response completeness for only one of the statements about screening coverage, components, and assessment methods. Participants who completed 10 or fewer statements were less likely to agree with the use of clinician preference and experience in the choice of neuro-behavioural assessment methods (42%) compared with participants who completed more than 10 statements (73%, χ^2^=7.2, p=0.007: Table 5, item 16).

## Discussion

This is the first systematic evaluation of health professionals’ perceptions of the need for and design of screening for FASD in Australia. We found consensus support for targeted screening, and more frequent agreement with screening in childhood than at birth. Although about half the participants supported the need for universal screening for FASD as an ideal and ethically responsible approach to minimise missed cases, consensus agreement was only achieved for targeted screening of individuals with relevant clinical presentations and groups at high risk of FASD. Parent or caregiver concern that their child may have a FASD and clinical presentations associated with the likelihood of prenatal alcohol exposure were most strongly endorsed as indications for targeted screening.

Targeted screening was considered more cost effective and feasible than universal screening, with support for targeted screening linked to constraints on service provision, including the need for provider training and increased demand for diagnostic and intervention services. Participants were also concerned about the limitations of existing screening methods and the need for programs to have acceptable sensitivity, specificity and cost-effectiveness. These findings reflect previously identified difficulties in screening for FASD [20,29,30], and deficits in diagnostic and management capacity to support screening [9,14,15,29,44,45].

We found consensus agreement on the need for a checklist and criteria for referral to support the implementation of screening for FASD. We also found consensus agreement that screening primarily requires health professionals to assess prenatal alcohol exposure and consider it as a possible cause of abnormalities. These findings are consistent with the need for greater awareness of FASD and improved capacity for screening among Australian health professionals [46,47], and highlight support for targeted screening as a strategy which can be integrated into usual clinical practice and passive case finding. Over half of participants agreed that most of the information required for FASD screening is assessed at birth or during relevant clinical presentations in childhood, which indicates some capacity for FASD screening in Australia. However, perceptions of participants in this study could differ systematically from health professionals who do not have experience or expertise in FASD screening or diagnosis.

Participants indicated that standard, explicit and efficient screening criteria are needed to determine when referral for a diagnostic evaluation is required. Few formal, validated instruments for FASD screening exist, and no single instrument is suitable for all ages and settings [29]. The absence of a single common distinguishing feature of FASD that can be used to indicate the need for a diagnostic evaluation has required programs to use aetiological risk factors and diagnostic features to determine whether a diagnostic evaluation is required. Although these screening methods have been considered unreliable, time consuming and rudimentary [48], most participants supported the use of screening indicators linked to aetiological and diagnostic factors, and emphasised the need to identify efficient and effective screening criteria.

Inaccurate FASD screening and case ascertainment have been linked to deficits in practitioner training and the use of inconsistent case definitions [8,26]. Formal assessment of facial dysmorphology and functional CNS performance at the screening stage was not considered necessary, reliable or appropriate in all settings. The need for valid population reference data for comparison was also identified. Although more than half of participants were not familiar with the formal assessment instruments for alcohol exposure presented, we found consensus agreement on the need for formal assessment methods, and on the usefulness of the AUDIT-C assessment instrument [41] following description of this instrument in round 2.

The facial photographic screening tool was considered to provide a feasible alternative to the measurement of facial anomalies by inexperienced assessors, although some participants indicated concerns about the use of facial dysmorphology assessment in FASD screening. While facial anomalies have been used to screen for FAS [26,27,29] facial anomalies are not commonly assessed in FASD screening [9,32]. The assessment of facial anomalies has been considered inappropriate in general population screening for FASD due to multiple factors, including its low predictive value for FASD [29]. However, diagnostic guidelines and criteria for referral recognise the significance of the characteristic FAS facial anomalies as an indicator of the need for diagnostic evaluation, particularly in the absence of confirmed prenatal alcohol exposure [6,9,12].

Criteria have been established to identify when population screening is appropriate and ethically justified, and to ensure that screening does more good than harm [49]. The variable application of these criteria in practice has been attributed to their inherent subjectivity and a lack of evidence to comprehensively evaluate screening interventions [50]. Criteria for the evaluation of population-based screening interventions include whether the potential impact of the condition is sufficient to justify screening; whether there is a benefit from early diagnosis and treatment; whether there is a cost-effective and acceptable screening test; and whether appropriate diagnostic services and treatment are available [49-51]. Our finding of a lack of consensus on the need for universal screening of apparently healthy individuals is consistent with the failure of FASD screening to meet some of these requirements.

Targeted screening for high risk presentations and groups was supported as a strategy to improve the identification of FASD in Australia, consistent with developments in health policy [23]. Participant comments emphasised the importance of ensuring the effectiveness of screening interventions prior to their use to ensure that the intervention benefits both individuals screened and the community. There is little reliable information on the epidemiology of FASD in Australia, and it is not clear what proportion of individuals with FASD would be identified by targeted screening of high-risk groups. Our findings highlight the complex issues that must be addressed when pursuing the deceptively simple objective of early diagnosis [49], and the real need for systematic evaluation of the risks and benefits of proposed screening interventions [50].

The Delphi method provides a strong basis for the construct validity of the study findings, with participants able to validate their initial responses and identify areas of uncertainty [34]. This study was primarily based on an exploration of established screening methods, and our use of non-representative sampling and a modified Delphi process may have limited our ability to evaluate all potentially relevant information on the design of FASD screening programs in Australia. Nevertheless, participants provided diverse perspectives on the use of screening for FASD, including the identification of risks associated with screening, and the importance of both individual case finding and a population based approach. Further research is needed to evaluate potential strategies to facilitate improved identification of FASD, and to evaluate their performance and acceptability in the Australian context.

We aimed to recruit health professionals who had specific experience or expertise in FASD screening or diagnosis to identify consensus perceptions on the approach to FASD screening in Australia. Paediatricians were most highly represented within the study sample, reflecting the profession’s key role in diagnosis in Australia. The round 1 response in this study exceeded the 70% recommended level [52] and was similar to that observed in other Delphi studies involving clinician panels [53,54]. Attrition was greatest between the time of agreement to participate in the study and returning the round 1 questionnaire, and similar to that reported by others [53]. Delay between recruitment of the panel and distribution of the questionnaire, as well as the recruitment of a large panel [55] may have reduced response in this study. Although we found response completeness was associated with participant occupation and experience, there was little evidence of differences in perceptions about screening according to response completeness.

Evaluation of pilot screening interventions are required to address the lack of evidence to support the effectiveness of screening for FASD in Australia and identify the impact of screening parameters on program outcomes and effectiveness. This approach to development would enable investigation of practical barriers to success, including the ability to engage with high risk groups. The development of effective screening interventions can improve our understanding of the epidemiology of FASD and its prevalence in high risk groups; contribute to the identification of appropriate strategies for FASD management and prevention; and provide an empirical evidence base for FASD policy in Australia.

It is clear from our findings that additional strategies are also required to improve passive case-finding, referral and diagnosis in Australia. Signs of FASD are frequently non-specific, and barriers to seeking or accessing appropriate diagnostic and intervention services are likely to be important obstacles to passive case finding. Strategies that can be used to improve passive case finding capacity and the identification of FASD in Australia include the development of resources and programs to improve awareness of the disorders and their prevention among health professionals, other relevant professionals and the wider community; the development of standard criteria to guide health professionals on appropriate referral of individuals who require specialist assessment, as implemented in North America [6,12]; and improved diagnostic capacity and access to specialist services.

## Conclusion

Our findings provide an agreed basis for the development and evaluation of targeted screening for FASD in high risk groups, standard criteria for referral, and other strategies to improve passive case identification, including improved awareness of the disorders among health professionals. Health professionals require well-defined, effective and ethically justified methods to identify and refer individuals who require specialist diagnostic assessment, and appropriate training to deliver these services. Established capacity for diagnosis and management is also essential, and the development of effective targeted screening interventions can contribute to the identification of appropriate strategies for the design and resourcing of FASD diagnosis, management and prevention services in Australia.

## Competing interests

The authors declare that they have no competing interests.

## Authors’ contributions

CB, EJE and JMP designed the study and CB and EJE supervised the study. CB, EJE, REW, JL and HJ designed the study questionnaire, and all authors, including consumer and community representatives, were members of a project steering group that reviewed the study methods and procedures. REW programmed the online questionnaire and analysed the data. REW drafted the manuscript and all authors edited the manuscript. All authors read and approved the final version of the manuscript.

## Pre-publication history

The pre-publication history for this paper can be accessed here:

http://www.biomedcentral.com/1471-2431/13/13/prepub
